# A novel protist parasite, *Salmoxcellia vastator* n. gen., n. sp. (Xcelliidae, Perkinsozoa), infecting farmed salmonids in Norway

**DOI:** 10.1186/s13071-021-04886-0

**Published:** 2021-08-28

**Authors:** Egil Karlsbakk, Cecilie Flatnes Nystøyl, Heidrun Plarre, Are Nylund

**Affiliations:** 1 Department of Biological Sciences, University of Bergen, PO Box 7803, 5020 Bergen, Norway; 2 Skodje, Norway

**Keywords:** Rainbow trout, Atlantic salmon, X-cells, Xcelliids, Perkinsozoa, Marine, Parasites

## Abstract

**Background:**

In Norway, x-cell parasites associated with disease in farmed salmonids have been known as a rare phenomenon for two decades. These parasites cause systemic infections in farmed rainbow trout (*Oncorhynchus mykiss*) and Atlantic salmon (*Salmo salar*), but have so far not been characterized and described.

**Methods:**

The x-cells from several cases of diseased fish were studied using light and electron microscopy, and by phylogenetic analysis based on small subunit ribosomal RNA (*SSU* rRNA) gene sequences.

**Results:**

We describe here the x-cell parasite as a new species in a new genus, *Salmoxcellia vastator* n. gen., n. sp. Phylogenetic analyses placed *Salmoxcellia* n. gen. together with *Gadixcellia* among the xcelliids, a group of perkinsozoan alveolates. The new genus and species were found to have vacuolate plasmodial x-cells filled with lipid droplets, and an electron-dense alveolar pellicle. Electron-dense cytoplasmic inclusions, which are characteristic of the other xcelliid genera *Xcellia* and *Gadixcellia*, are lacking in *Salmoxcellia* n. gen. These x-cell plasmodia divide by plasmotomy and occur as aggregates in the host tissues, particularly in blood-rich tissues such as those of the kidney, red musculature, heart and liver. Host reaction and the refractive lipid droplets in the x-cells result in *S. vastator* n. gen., n. sp. aggregates appearing as white patches in the tissues.

**Conclusions:**

We describe a new genus and species of xcelliid protist parasites from two very important farmed fish species and provide molecular methods for detection. The new parasite is associated with disease, but more importantly it has a spoiling effect on farmed salmonid fillets, rendering them unsuitable for sale. Consequently, this parasite represents a threat to the aquaculture industry.

**Graphical abstract:**

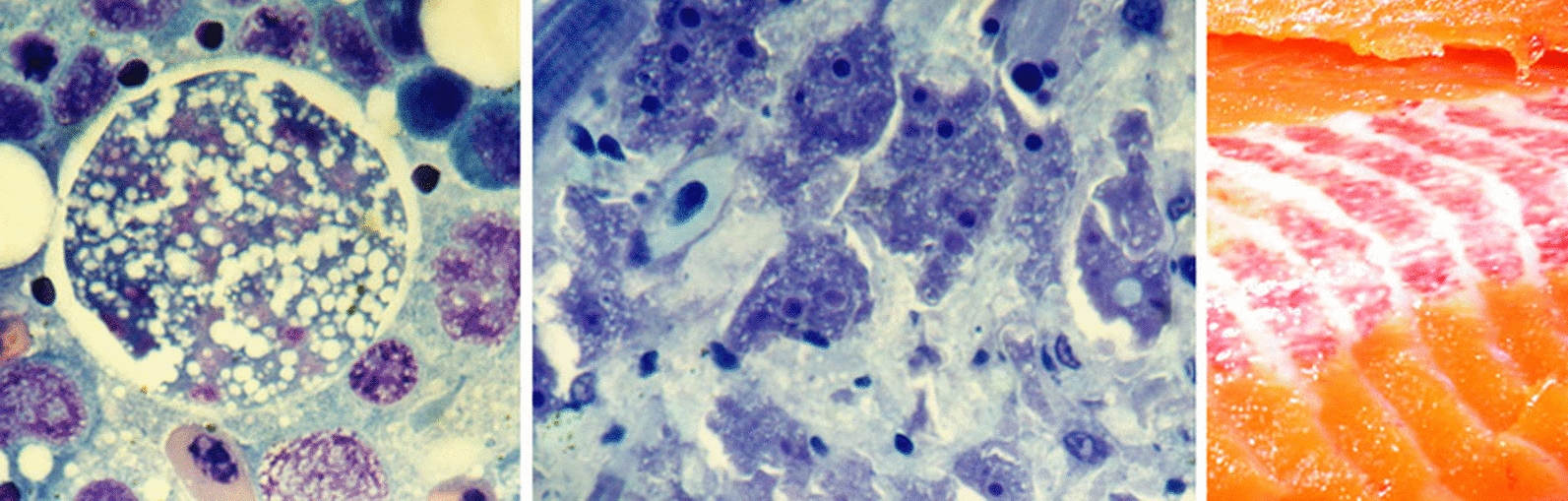

## Background

A farm with seawater netpen-reared adult rainbow trout (*Oncorhynchus mykiss*) in western Norway experienced increased mortality during the autumn 2002, and at slaughtering at the end of the year a high number of fishes could not be sent to the market due to poor quality of the filet. Affected fish showed extensive whitish nodule-like lesions in most tissues and organs, including the musculature [1, 2]. These systemic lesions were found to be associated with aggregates of multinuclear cells (‘x-cells’), containing refractive granules. This disease also affected rainbow trout production in 2003 but was subsequently not detected again until 2018–2019 when it was detected in several rainbow trout farms in western Norway. The disease continued to be detected into 2020 at which time it was also detected in a salmon broodfish population containing large mature salmon.

In 2002, a small subunit ribosomal RNA (*SSU* rRNA) gene sequence was obtained from the nodules with x-cells, showing that these cells represented a protist parasite then found to have affinity to members of the genus *Perkinsus* (class Perkinsea), a group of important shellfish pathogens [1, 2]. This novel perkinsean sequence was always obtained from salmonids infected by the x-cell parasite.

X-cells have been found in several fish species and are usually associated with the pseudobranch, skin or gill lesions. Recently obtained sequences show that these are also related to genus *Perkinsus* [3–5]. Two genera have been characterized, *Gadixcellia* and *Xcellia*, in the family Xcelliidae, representing a sister group to *Perkinsus* spp. among the other perkinsozoans. *Gadixcellia* spp. cause pseudobranch-associated xenomas in certain gadid fishes, while the *Xcellia* spp. are gill and skin parasites of flatfish, eelpouts, icefish and gobies in North Atlantic, North Pacific and Antarctic waters [5].

A major difference between the Norwegian salmonid x-cell parasite and *Gadixcellia* spp. and *Xcellia* spp. is that the former causes systemic infections. One study reported the presence of x-cells in an internal organ of a salmonid, namely multinuclear x-cells in the liver of coho salmon (*Oncorhynchus tshawytscha*) cultured on the coast of Galicia, Spain [6].

Here we describe the x-cell parasite infecting farmed rainbow trout and salmon in western Norway as a new xcelliid species in a new genus, based on cell morphology, *SSU* rRNA gene sequences and phylogenetic position, and also report observations on the tissue trophism of the parasite and the disease it is associated with.

## Methods

### Cases and fish samples

Samples from infected fishes were obtained from four cases (A–D), as described in the following sections.

#### Case A

During the autumn of 2002, a low mortality rate was observed among rainbow trout stocked January 2001 in a fish farm in Nordfjord, western Norway associated with a pathology not previously observed. Increased mortality was noted in September at a sea temperature of 15 °C, and the accumulated mortality reached 2% by harvest in December. At harvest there was a high discard rate due to poor filet quality (spotted flesh). The affected fish weighed between 2 and 3.5 kg. Samples were obtained from six individual fish collected on 6 November and one fish collected on 21 November. These samples were used for nucleic acid extraction, parasite SSU rDNA sequencing and histology studies.

#### Case B

The same disease as in case A was observed in another rainbow trout (Case B) cohort in December 2003 in the same region as case A. Samples were taken from two fish (aprox. 0.7 and 0.9 kg, respectively) for histology studies, molecular testing and SSU rDNA sequencing of the parasite.

#### Case C

Rainbow trout from a seawater fish farm in Hordaland, western Norway showed white nodules in the internal organs. Two fish (weight between 0.3 and 0.4 kg) were examined on 21 February 2019. Nodules and random tissues were sampled and analysed; parasite SSU rDNA was extracted and used for sequencing studies.

#### Case D

During a disease outbreak (likely cardiomyopathy syndrome) at an Atlantic salmon (*Salmo salar*) broodstock farm in western Norway in 2020 white spots were observed in some organs and tissue samples (gills, liver, heart and red muscle) of some of the 31 mature brood fish sampled. Most of these fish were deep-frozen and examined in the thawed condition. Samples were taken for molecular testing, sequencing of SSU rDNA and histology studies.

### Histology

Tissues, gills, somatic muscle, liver, heart, kidney, spleen and hindgut from rainbow trout and salmon suffering from this disease at marine production sites were fixed in a modified (phosphate-buffered) Karnovsky fixative and stored at 4 °C before processing for histology studies [7]. The tissues were either embedded in EMbed 812™ (Electron Microscopy Sciences, Hatfield, PA, USA) and semi- and ultrathin sections cut from the resin blocks for used in light- and transmission electron microscopy (TEM) studies (see [7]), or embedded in Historesin™ (Leica Microsystems, Wetzlar, Germany) and semithin sections cut and stained with either toluidine blue or Hemacolor™ (Merck & Co., Kenilworth, NJ, USA).

### DNA/RNA extraction and sequencing

RNA was extracted as described by Gunnarsson et al. [8], and DNA was extracted using the DNeasy DNA Tissue kit (Qiagen, Hilden, Germany) as recommended by the manufacturer. DNA was diluted twice in 50 μl 10 mM Tris–HCl (pH 8.5) to increase the overall DNA yield. RNA and DNA were stored at − 50 °C until use. The extracted RNAs were used for real-time reverse transcriptase (RT) PCR for detection of the parasite in different tissues from both fish species.

The DNA extracted from tissues showing pathological changes was used for SSU ribosomal DNA (SSU rDNA) gene sequencing of the parasite. Sequences were obtained with the 18c or PerF2 forward primers and the reverse primers RosR2 or RosR3, or the forward primer RosF2 and the reverse primers 1492R, RosR2 or UnR. The primers are given in Table 1.

**Table 1:** Primers used to obtain partial small subunit rRNA gene sequences of the salmonid x-cell parasite, and primers and probe of the real-time PCR assay with Per primers

Primer name	Sequence (5′–3′)	Position^a^
18c	TGGTTGATCCTGCCAGT	Outside, 5′ end
PerF2	TTCTGACCTATCAGCTATGGACG	280–302
RosF2	CCATAAACTATGCCGACTAGG	1019–1039
RosR2	ATCCTTCCGCAGGTTCACCTACGG	Outside, 3′ end
RosR3	TAGTCGGCATAGTTTATGGTTAGG	1037–1014
1492R	TACGGYTACCTTGTTACGACTT	Outside, 3′ end
UnR	GACGGTATCTGATCGTCTT	1008–990
PER-F	CCCTGCTAAATAGTATGCGGTATACA	1332–1357
PER-R	ACCTTCAAAATAAGAACAATCAGCAA	1402–1377
PER-probe	6FAM-AGCGTTGAGCGGAT-MGB-NFQ	1373–1360

^a^Positions are with reference to the MW743278 sequence

### Real-time RT-PCR

A real-time RT-PCR (henceforth ‘qPCR’) assay (‘PER-assay’) was developed targeting the *SSU* rRNA of this new x-cell parasite, with the forward primer PER-F, the reverse primer PER-R and the Taqman PER-probe (Table 1). Product size was 71 bp, and the efficiency *E* = 10 [− 1/slope] [9] was 1.98. A previously developed assay (EF1A, *E* = 1.94, Olsvik et al. [10] targeting the elongation factor 1 alpha mRNA, was used to ascertain RNA quality (extraction control). Slopes were estimated from standard curves for the assays generated by tenfold RNA dilutions in triplicates.

### Phylogenetic analyses

The phylogenetic affinities of the x-cell parasite, based on the *SSU* rRNA gene sequence (1860 bp), was first examined by searching (BLASTn, megaBLAST) the GenBank database for related homologous sequences using BLAST (2.0). Sequences from described species, as well as some environmental sequences, were aligned (AlignX, Vector NTI Suite software), and the alignment edited in GeneDoc (www.psc.edu/biomed/genedoc) to allow comparison of homologous positions. Maximum likelihood trees were generated and bootstrapped (50,000 puzzling steps) in TREE_PUZZLE 5.2 (http://www.tree-puzzle.de). Phylogenetic trees were drawn using FigTree (v1.4.3, released by A. Rambaut: https://github.com/rambaut/figtree/releases).

## Results

### Gross pathology

**Fig. 1:**
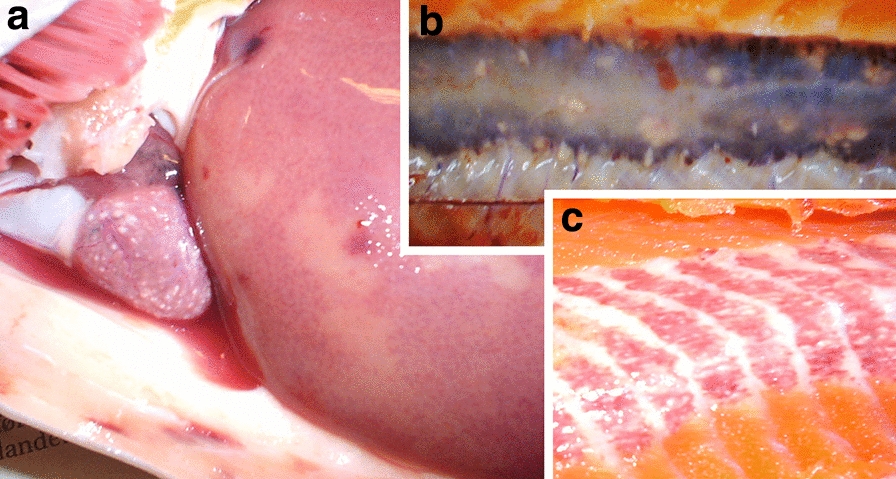
Diseased rainbow trout (*Oncorhynchus mykiss*) showing macroscopic signs of salmoxcelliosis. **a** Whitish spots in heart and blotched liver, **b** pale nodules in the kidney, **c** white-spotted red muscle

Diseased rainbow trout (cases A and B) typically showed white spots (nodules) and petechial bleedings in the viscera and on the peritoneal covering of the visceral cavity. Some fish showed excess ascitic fluid. The heart, particularly the ventricle, was partly or completely covered with white spots (Fig. 1a) that merged into a thick whitish covering in advanced cases. The liver was typically blotched with pale yellowish areas (Fig. 1a), at the centre of which there could be areas of bleeding. There were few distinct white spots or patches in the liver. The kidney could be enlarged and could contain large numbers of whitish nodules, but this organ was usually less affected than the heart and the red musculature (Fig. 1b). The spleen was also often enlarged, but did not contain visible nodules. Gonads contained a moderate numbers of nodules. There were generally few nodules in the intestinal wall, but some individuals had gravely affected hindguts. In the musculature, the red muscle was particularly affected, with high densities of whitish lines in between the muscle fibres (Fig. 1c) that occasionally contained melanin. The the white muscle had were fewer whitish lines, but numerous focal bleedings could occur. The white nodules were also seen in the skin.

**Fig. 2:**
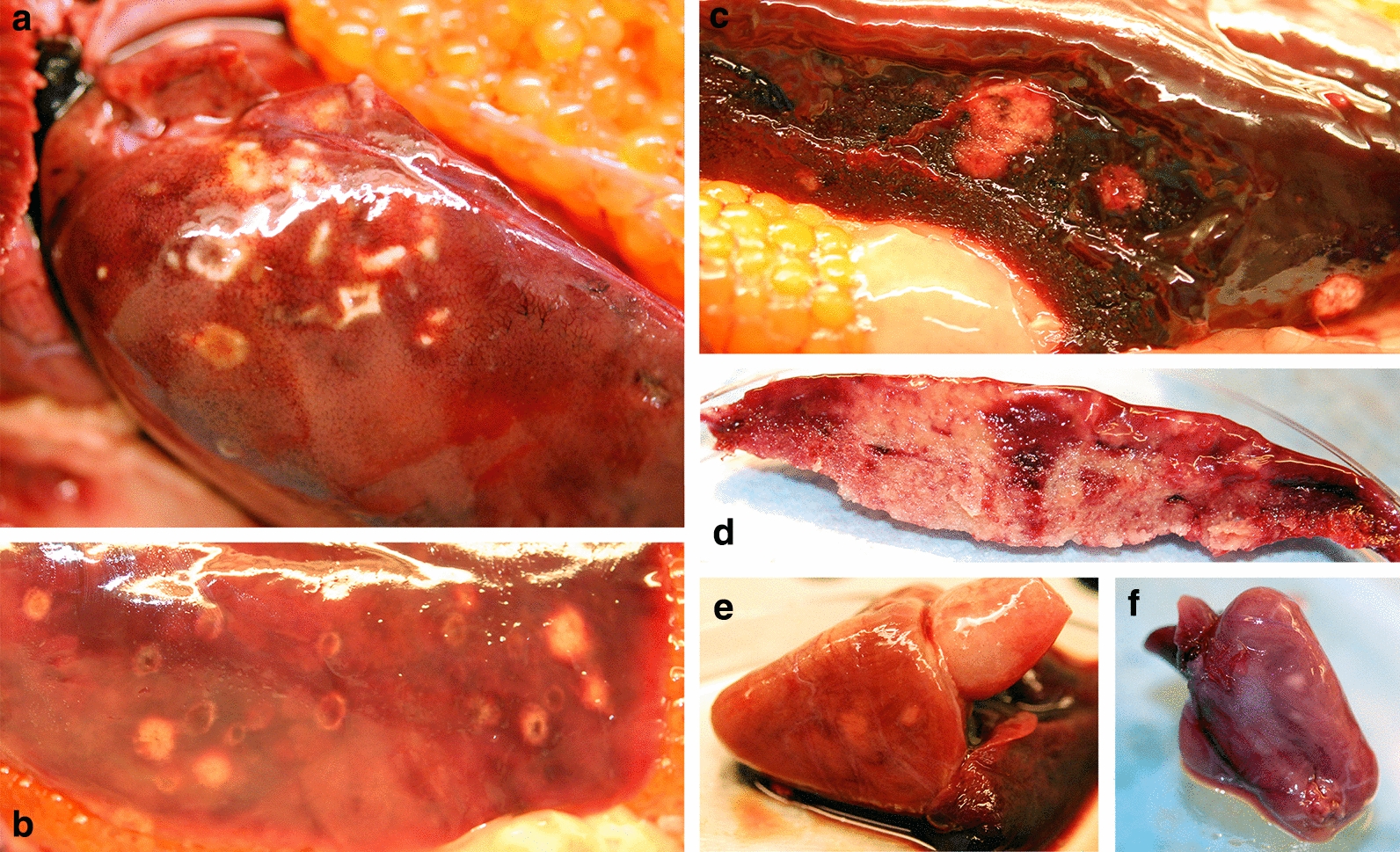
White patches in liver and heart tissues of Atlantic salmon (*Salmo salar*) broodstock, representing x-cell aggregates. **a**, **b** Ring-like spots on liver (arrow), **c** appearance of spot inside liver, **d** heavily infected liver with pale sections when cut, **e**, **f** pale areas in heart ventricle

The Atlantic salmon broodstock (case D) showed whitish patches in the liver, heart and kidney of seven, four and two of the 31 specimens examined, respectively (Fig. 2). No whitish patches were seen in the red musculature of the salmon. However, screening with qPCR revealed that 23 specimens were positive for parasite RNA. The corresponding tissue distribution of the x-cell parasite as revealed by qPCR is shown in Table 2.

**Table 2:** Tissue distribution of lesions and quantitative PCR-positive samples from Atlantic salmon (*Salmo salar*) broodstock (*n* = 31)

Organ	No. of fishes with lesions	No. of positive fish by qPCR	*Cq* value range (mean)^a^
Liver	7	10	10–31 (18)
Heart	4	14	10–35 (24)
Kidney	2	14	19–35 (26)
Red muscle	0	23	12–33 (23)
Gill	0	16	16–37 (27)

^a^Cycle threshold (*Cq*) values; lower *Cq* values reflect higher amounts of target RNA

Whitish areas of the liver gave low Cq values, while apparently normal parts were negative or had high Cq values.

### Taxonomic description

**Class Perkinsea Levine, 1978**

**Order Perkinsida Levine, 1978**

**Family Xcelliidae** Freeman, Fuss, Kristmundsson, Keeling, Shalchian-Tabrizi & Bass, 2017 (emend.)

Parasitic alveolate cells found in tumour-like aggregations (xenomas) or dispersed in the tissues of marine teleosts. Cells have a polygonal or irregular appearance in histological sections, can be large (> 20 μm) and contain one to many weakly-staining nuclei, each with a prominent nucleolus. X-cells are free, but occasionally surrounded by enveloping host cells. Ultrastructurally, xcelliids have a plasmalemma thickened by electron-dense material that may be subtended by endoplasmic reticulum (ER) cisternae producing a three-layered alveolar pellicle. X-cells have mitochondria with limited inner membrane folding; cristae, when present, are tubular.

**Genus** ***Salmoxcellia*** **Karlsbakk, Nystøyl, Plarre & Nylund, n. gen.**

**Diagnosis:** Multinuclear x-cells (plasmodia) producing systemic infections in salmonid fishes. Alveolar pellicle with several electron-dense membranes. Invaginations producing deep clefts in cytoplasm. Cytoplasm vacuolate, lipid droplets dominating. Nuclei with prominent nucleoli. Mitochondria sparse, with few tubular cristae. *Salmoxcellia* n. gen. differ from other xcelliid genera by lacking electron-dense cytoplasmic granules.

**Etymology:** The genus is named after the host family Salmonidae (*Oncorhynchus mykiss* is non-native so *Salmo salar* is the only known native host) and Xcellia (generic name).

**ZooBank registration:** The LSID for the new genus is XXXX

***Salmoxcellia vastator*** **Karlsbakk, Nystøyl, Plarre & Nylund, n. sp.**

***Type-host***: *Oncorhynchus mykiss* (Salmonidae), rainbow trout.

***Other hosts***: *Salmo salar* (Salmonidae), Atlantic salmon.

***Type-locality***: Fjordane, northern part of Vestland county, western Norway.

***Other localities***: Hordaland, Vestland county, Norway.

***Type-specimens***: Ethanol sample, pieces of heart and liver of a heavily infected rainbow trout from case A (ZMBN 140945) and formalin fixed tissues, now in 96% ethanol of a heavily infected rainbow trout from case A (ZMBN 140946) deposited in Zoological Museum, University of Bergen, Norway.

***Site of infection***: Systemic, but mainly in heart, red musculature, kidney and in liver.

***Representative DNA sequences***: Partial sequences of *SSU* rRNA gene were submitted to the GenBank database under the accession numbers MW743278–MW743281.

***ZooBank registration***: To comply with the regulations set out in article 8.5 of the amended 2012 version of the International Code of Zoological Nomenclature (ICZN) [11], details of the new species have been submitted to ZooBank. The Life Science Identifier (LSID) of the article is urn:lsid:zoobank.org:pub:1A3C284E-2331-4C27-9F9E-8005525DE40E. The LSID for the new genus *Salmoxcellia* is urn:lsid:zoobank.org:act: 029F9F43-0762-409C-975D-91F56B80C3E2. The LSID for the new species *Salmoxcellia vastator* is urn:lsid:zoobank.org:act: 6438C2EB-23C0-4768-9A85-B2DF3DA18D27.

***Etymology***: The species is named after the spoiling effect it has on the fillet appearance (seafood quality) of farmed salmonids at harvest [vastator (L.), noun, destroyer].

### Description

#### Morphology

**Fig. 3:**
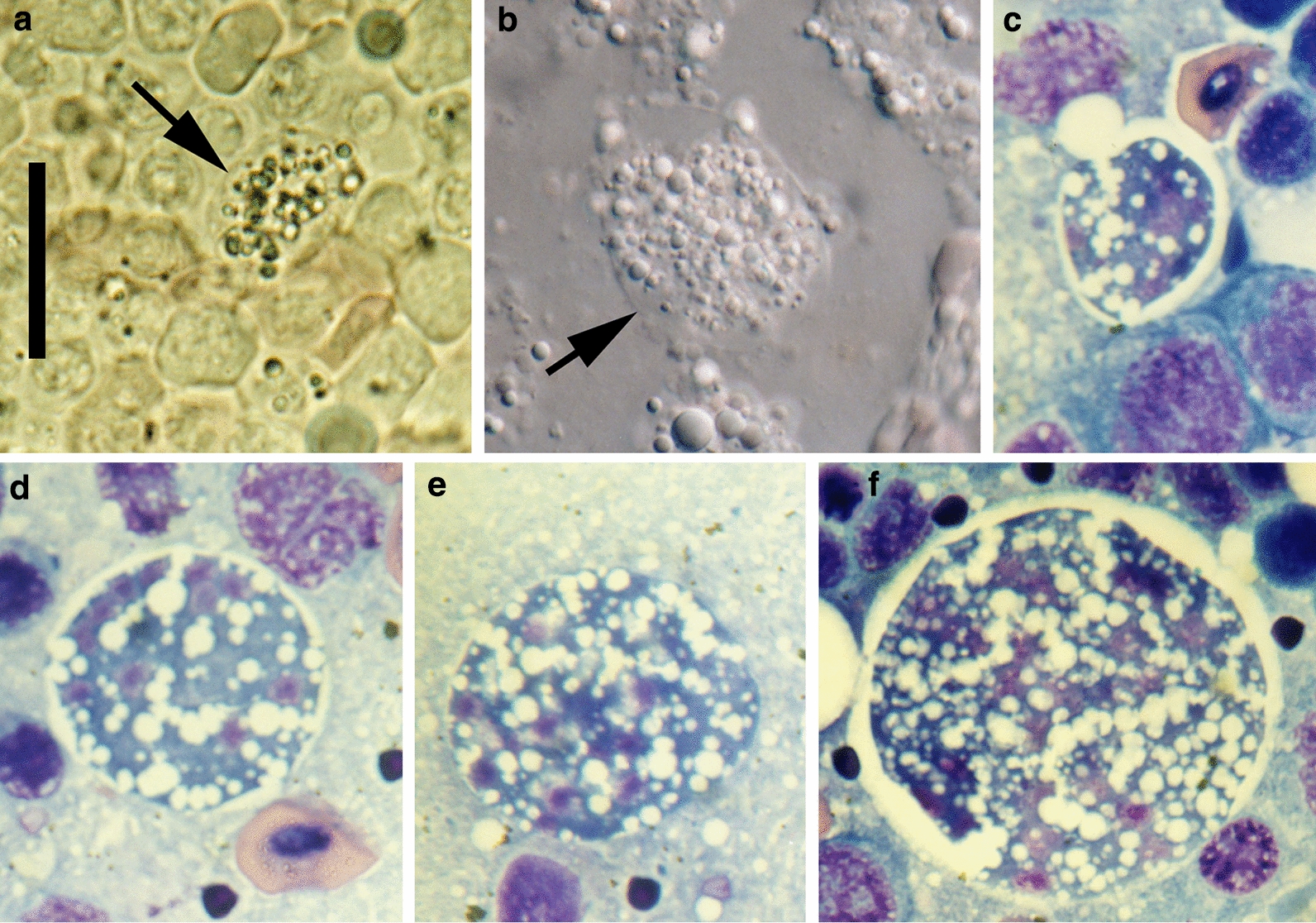
X-cells from rainbow trout kidney. **a**, **b** Squash preparations, showing x-cells (arrows) filled with refractive granules (**a** brightfield, **b** interference contrast). **c**–**f** X-cells in Hemacolor-stained kidney imprints. Note multiple nuclei and vacuolate cytoplasm; methanol fixation removed the refractive granules. Scale bar (**a**–**f**): 20 μm

Microscopic examination of fresh squash preparations of pale parts of the liver or of whitish nodules from other organs of the affected rainbow trout revealed numerous large cells (mostly 15–40 µm in diameter) filled with refractive granules. These cells (‘x-cells’) were more rigid and the cell membrane appeared more distinct than in other cells (Fig. 3a, b). The x-cells were not seen in liver parts of normal colouration, or in tissues without spots/nodules. The highest numbers occurred in imprints from affected heart, red muscle and kidney. The parasite was not observed in the brain.

**Fig. 4:**
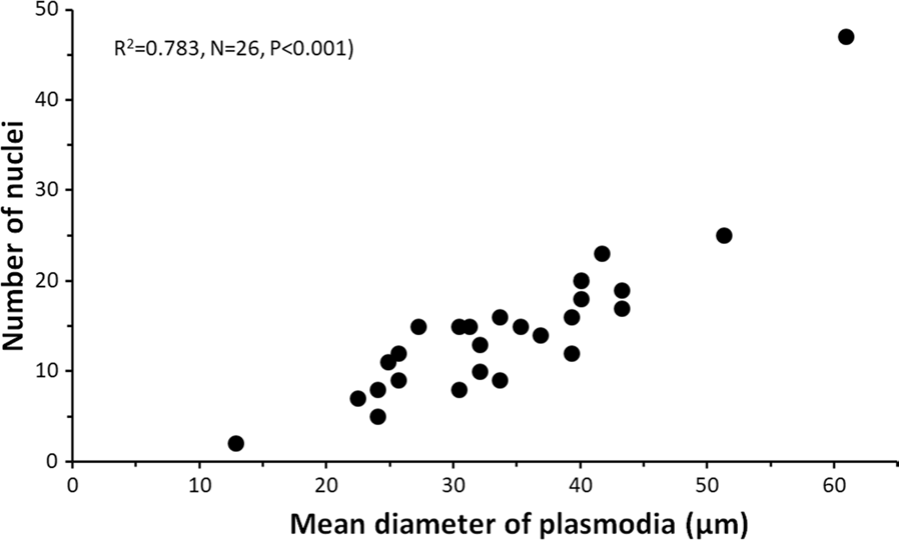
Relationship between the size of the multinuclear x-cells (plasmodia) in stained kidney imprints and the number of nuclei counted (Pearson *R*^2^ shown)

In stained tissue imprints, these x-cells were found to be multinuclear (i.e. plasmodia). They were highly vacuolate, rounded and variable in size (mean diameter 33 μm, range 12–61 μm; *n* = 59) and with several nuclei (median 12, range 2–47, mode 15; *n* = 35) (Fig. 3c–f). There was a highly significant positive relationship between the diameter of the plasmodia and number of nuclei (Fig. 4). A very large plasmodium (approx. diameter 61 μm) was bilobed, apparently dividing by plasmotomy.

**Fig. 5:**
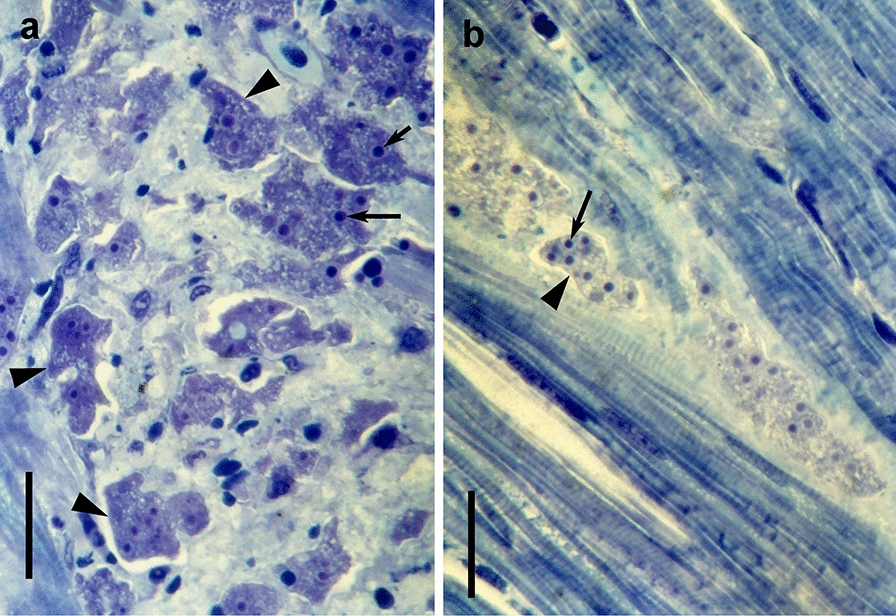
X-cell plasmodia in the red musculature of rainbow trout. **a** Congregation of plasmodia in cellular debris. **b** Plasmodia arranged in a line along muscle fibers. The plasmodia (arrowheads) harbour several pale nuclei with strongly stained karyosomes (arrows). The scale bars are 20 μm

Aggregations of the parasite were observed in histological sections of white spots, nodules or areas from the various tissues (heart, liver, kidney, musculature) (Fig. 5). These were irregular in outline and granular and usually had a strongly stained cytoplasm. Nuclei were pale, but with strongly stained karyosomes.

Adjacent tissues and cells could have a normal appearance, but an acellular matrix was present around large x-cell aggregations that could contain necrotic cells with pyknotic nuclei and some mononuclear leukocytes.

**Fig. 6:**
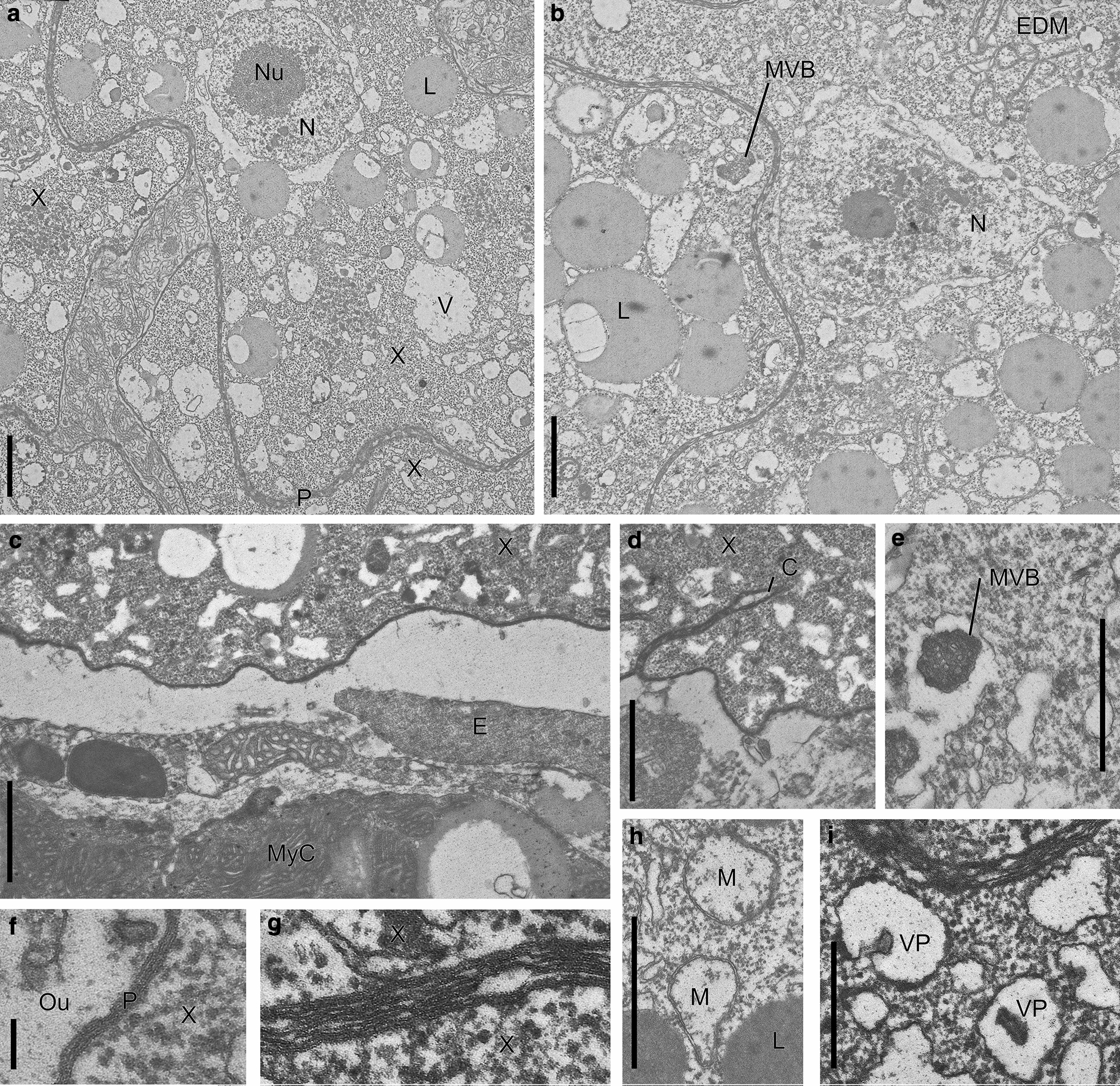
Ultrastructure of x-cell plasmodia from rainbow trout. **a**, **b** Vacuolate x-cells (*X*) with marked (electron-dense) adjoining pellicles (*P*), lipid droplets (*L*), nucleus (*N*) with marked nucleolus (*Nu*) and vacuoles (*V*). In **b**, a multivesicular body (*MVB*) and a region in the cytoplasm with an aggregate of electron-dense membranes (*EDM*) are visible. **c** Part of intravascular parasite (*X*) close to the endocard endothelium (*E*).* MyC* myocardial cell. **d** Deep pellicle-covered invagination into the x-cell, interpreted as a cleft (*C*). Some invaginations appeared to be branched. **e** Multivesicular body (*MVB*) inside a vacuole, apparently confluent with that of another MVB. **f** Detailed view of pellicle (*P*) normally composed of three membranes.* Ou* Outside. **g** Contact point between two x-cells, where eight membranes can be counted. **h** Mitochondria with no visible cristae. **i** Vacuoles with vacuoplast (*VP*)-like electron-dense inclusions. Scale bars: 1 µm (**a**–**e**, **h**; 0.1 µm (**f**–**g**; 0.5 µm (**i**)

Ultrastructurally, the x-cells were readily distinguished from host cells by their thick electron-dense pellicle, several nuclei and a cytoplasm filled with lipid droplets and vacuoles. Nuclei measured 2.3–4.5 µm in diameter and had a large nucleolus (diameter: range 0.7–2.2 μm, typically 1.6 µm) (Fig. 6a, b). Lipid droplets were generally present in large numbers, typically measuring 1.2 (range 1–1.9) µm in diameter (Fig. 6a, b), but a few cells contained fewer and smaller droplets (diameter: range 0.6–1.0 µm). Vacuoles were smaller than the lipid droplets (diameter: range 0.7–0.9 µm), and often contained an electron dense invagination or vacuoplast-like structure (Fig. 6a, i). The cytoplasm was dense with ribosomes, and rough endoplasmatic reticulum was generally present. Occasionally thread-like electron dense ER-cisternae were seen (Fig. 6b), confined to a certain part of the cell. These could reach 1.6 µm in length, and were 16–18 nm thick. Mitochondria were sparse, round or ovoid (diameter: mean 0.40 μm, range 0.23–0.58 µm; *n* = 31) and mostly confined to central parts of the cell. Small mitochondria had few tubular cristae, while larger mitochondria showed no cristae (Fig. 6h). Multivesicular bodies inside a dilated vacuole were common (Fig. 6b, e), with up to eight seen in a section of a single x-cell. The thick (mean thickness: 28 nm, range 20–43; *n* = 23) electron-dense pellicle normally consisted of three membranes (Fig. 6f), but occasionally two or four were seen. Six to eight membranes were observed at the border between adjacent x-cells (Fig. 6g). Deep invaginations of the cell pellicle were seen (Fig. 6d), reaching a depth of 3 µm. However, no micropyles or clear evidence for exo-or endocytosis were observed.

Host cells in the close vicinity or in contact with the x-cells could be intact and have a normal appearance (Fig. 6c). However, x-cell aggregates in the skeletal muscle and liver were free in a matrix abundant in free collagen fibrils. Immune cells were rarely seen.

### Remarks

The new genus and species differ from all other xcelliids (genera *Xcellia* and *Gadixcellia*) by showing multinuclear vacuolate plasmodial x-cells, with a pellicle composed of several electron-dense membranes. Electron-dense cytoplasmic inclusions, a characteristic feature of the other xcelliid genera *Xcellia* and *Gadixcellia*, are lacking in *Salmoxcellia* n. gen. Unlike the members of genera *Xcellia* and *Gadixcelli*, the new genus and species produce systemic infections in the fish host.

The new genus and species are also readily identified by a unique* SSU* rRNA gene sequence, and a distinct phylogenetic position.

### Sequences and phylogeny

A partial *SSU* rRNA gene sequence from the x-cell parasite was first obtained from the red musculature in the tail region of a rainbow trout in 2002 (GenBank: MW743279). The sequence then showed highest identity (82.7%) with members of the genus *Perkinsus* (*P. andrewsi*, GenBank: AF102171), hence the parasite was previously referred to as a perkinsean [1, 2]. In 2002, 2003, 2019 and 2020, identical sequences (1818 nt compared) were obtained from rainbow trout and Atlantic salmon harbouring x-cells (GenBank: MW743278-MW743281). In BLASTn searches, these sequences show the highest identity (88.3%) with *Gadixcellia gadi*, an x-cell parasite infecting cod *Gadus morhua.*

**Fig. 7:**
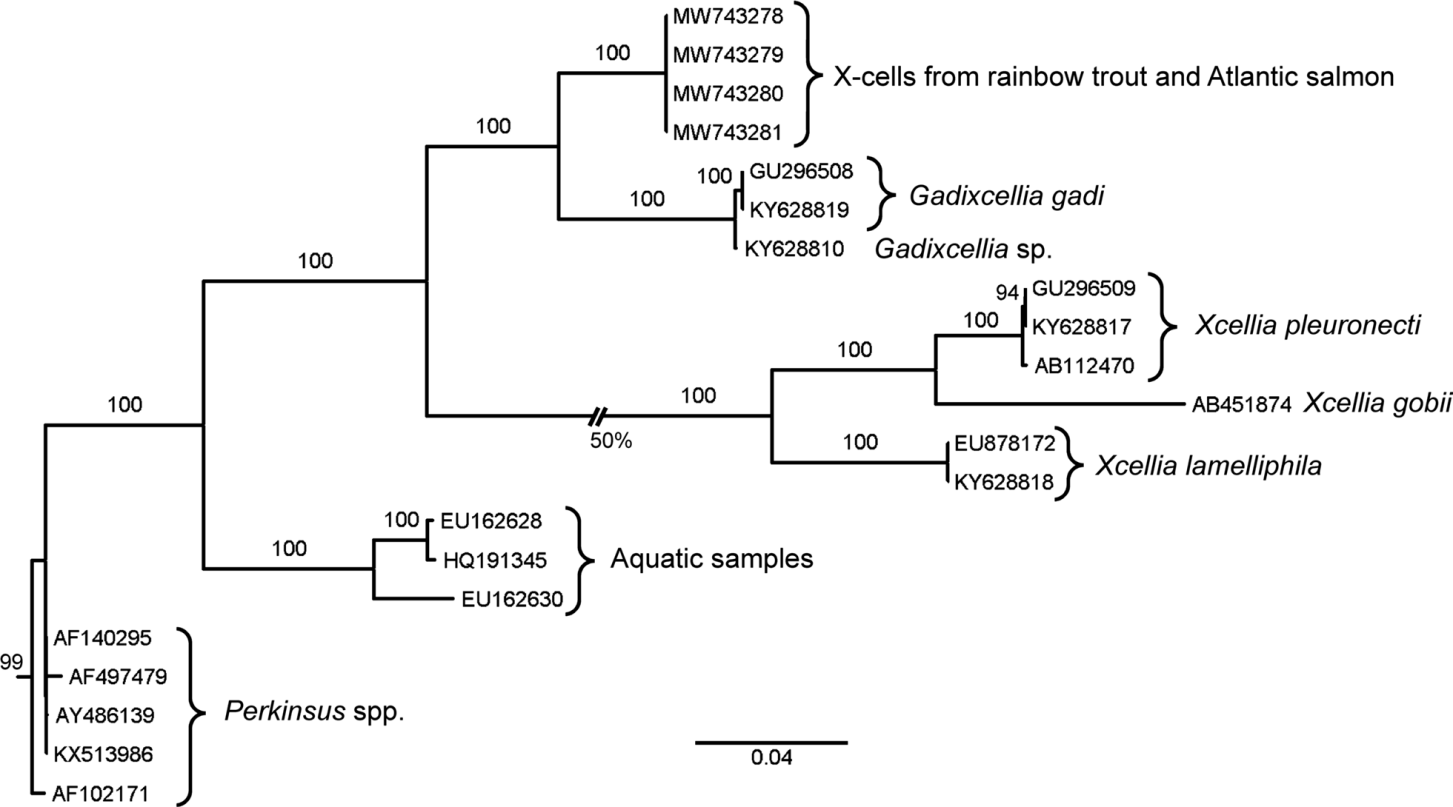
Phylogenetic relationship between the salmonid x-cells (*Salmoxcellia vastator* n. gen., n. sp.) and the related xcelliids and Perkinsea based on small subunit rRNA gene sequences. Maximum likelihood quartet puzzling tree. Scale bar: 4% estimated sequence divergence. Numbers at nodes: Tree-Puzzle ( (http://www.tree-puzzle.de) support values

Phylogenetic analyses showed that the closest relatives to the salmonid x-cell parasite are *Gadixcellia gadi* and *Gadixcellia* sp. infecting the blue whiting *Micromesistius poutassou* (Fig. 7). The closest sister group to the *Gadixcellia* branch that include the sequences of the present salmonid x-cells is constituted by *Xcellia* spp.

## Discussion

The term ‘x-cells’ were first used for a number of abnormal cells that occurred in epidermal tumours of some US Pacific flatfish [12]. Subsequently, ultrastructurally similar cells observed in skin or gill lesions in Pacific and Atlantic flatfish or in gadid pseudobranch-associated tumour-like growths have also been referred to as x-cells. These cells are regarded as neoplastic cells [13, 14], cells transformed by a viral infection (see [15]) or as a protist parasite (e.g. [16, 17]). The first proof that such x-cells were indeed a protist parasite was provided by Miwa et al. [18], who obtained a protist *SSU* rRNA gene sequence of x-cells from the Japanese flatfish *Hippoglossoides dubius*, thereby confirming the association with x-cells using* in situ* hybridization. Subsequent studies have corroborated this link for other x-cell parasites in other hosts [3, 19]. Initial analyses based on x-cell *SSU* rRNA gene sequences (now *Xcellia* spp.) showed that parasites from different hosts were related to each other, but revealed no clear phylogenetic placement of these parasites among the protist groups [18, 19]. The addition of more sequences, including the parasite later named *Gadixcellia gadi*, further supported a distinct phylogenetic grouping of these parasites, placed within the alveolates [3, 4]. Phylogenomic analyses confirmed the alveolate affinities and suggest a sister group relationship with Perkinsea (*Perkinsus*) in the Dinozoa [5].

Based on *SSU* rRNA gene sequences, *Salmoxcellia vastator* n. gen., n. sp. is most closely related to *Gadixcellia gadi*, and the genera *Gadixcellia* and *Salmoxcellia* are sister groups, rather distantly related to genus *Xcellia*. We do not concur with Freeman et al. [18] that *Xcellia*, *Gadixcellia* and now also *Salmoxcellia* should be considered to be members of family Xcelliidae. The xcelliids are a morphologically and phylogenetically distinct grouping that could be considered a class with two families.

No xcelliid life-cycles are known. Transmission experiments with *G. gadi* and *Xcellia* spp. have been unsuccessful [4, 5]. We have observed high concentrations of *G. gadi* cells in the urine of cod with pseudobranchial x-cell pseudotumours, suggesting that dispersal to the environment can occur* via* that route. It seems possible that *S. vastator* n. gen., n. sp. also could be released* via* urine in the kidneys or* via* bile in the liver, but since the infections are systemic the way of exit from the host is enigmatic. To date, all cases are from salmonids reared in seawater, and indeed most xcelliids are marine. It is possible that there is another host or environmental stage involved in the life-cycles [4, 5].

Most cases of salmoxcellosis are from rainbow trout, a non-native species in Norway. Also, the similar x-cell parasite observed in Spain [6] was in a non-native salmonid, coho salmon. Hence these x-cell parasites in *Oncorhynchus* spp. could infect and exploit non-native and possibly naïve hosts, causing disease. The only known enzootic host to *S. vastator* n. gen., n. sp. is Atlantic salmon, which could be a natural host. However, healthy farmed and wild Atlantic salmon have not been found to be infected (unpublished observations). The development of salmoxcelliosis in Atlantic salmon must be a very rare occurrence, considering the volume of farmed Atlantic salmon produced in Norway and the rarity of cases. The present case represented large and sexually mature broodstock, with the possibility that debilitation, another disease or immunosuppression could play a role in allowing parasite proliferation.

In the present cases, x-cells occurred in large numbers in white patches in diseased farmed fish. When alive, the x-cells seemed to be packed with refractive granules; these are removed by alcohol treatment, suggesting they contain lipid. Ultrastructurally they are seen as lipid vacuoles. The refractiveness of these lipid-filled x-cells is likely the main cause for the whitish appearance of x-cell aggregates, here referred to as patches or spots. They are not true granulomas; the x-cell aggregates are seen in a matrix composed of collagen, but in our rainbow trout cases there were few intact fibroblasts and leukocytes. However, in several instances, apparently unaffected host cells such as myocytes or endothelial cells were in close contact with the x-cells. A histopathology study of salmoxcelliosis is ongoing and will appear elsewhere.

The main sites of x-cells are blood-rich tissues and organs, such as heart, kidney and red musculature, but most tissues contain x-cells or are qPCR positive for parasite RNA. This could reflect a spread* via* blood, i.e. a systemic infection. Indeed, our ultrastructural studies on the heart revealed x-cell plasmodia attached to heart endothelia. Also, the gills are often qPCR positive in infected fish, likely due to the presence of intravascular parasites that could allow the development of non-lethal (gill biopsy based) methods of detection. In the liver, also a major site, the parasite is seen to be histozoic in groups but often more dispersed than in the patches of the musculature, kidney or heart.

Ultrastructurally, *Salmoxcellia vastator* n*.* gen., n. sp. seems to be very similar to the x-cells observed propagating in the liver of a coho salmon in Spain [6]. That parasite was compared to cod x-cells [16], a parasite now named *Gadixcellia gadi* [5]. We believe that the coho parasite and *S. vastator* n. gen., n. sp. are congeneric, and likely conspecific. The only difference is the observation of apparent endocytosis in the coho parasite [6], which is never seen in rainbow trout x-cells. The salmonid parasites differ from members of the genera *Gadixcellia* and *Xcellia* by being multinuclear, showing deeper invaginations of the outer membrane and lacking electron-dense bodies in the cytoplasm. *Xcellia* spp. have one or—rarely—two nuclei [3, 20], while *Gadixcellia gadi* is normally mononuclear but can produce plasmodia with up to eight nuclei [5, 16]. The presence of invaginations is also a trait in common with *G. gadi* and *Xcellia* spp. [3, 16, 21], but in these species they are shorter (< 1.1 µm in *Xcellia* spp., < 1.9 µm in *G. gadi*). The continuous EM profiles of these invaginations in the salmonid x-cells suggest that these are in fact clefts. Therefore, they could also represent partitions of the cytoplasm that eventually could be involved in plasmotomy. Large *S. vastator* n. gen., n. sp. plasmodia apparently divide by plasmotomy, as observed in the present study in stained kidney imprints. Division is poorly known in the xcelliids. Amitotic nuclear division occurs in the goby *Acanthogobius flavimanus* skin x-cells (likely *Xcellia gobii*) prior to cytokinesis, producing cells with two or sometimes more nuclei [17]. Similar observations have been done on dab (*Limanda limanda*) x-cells (likely *X. lamelliphila*), albeit rarely [3, 20].

The presence of cytoplasmic electron-dense membrane-bound granules is a shared character of genera *Gadixcellia* and *Xcellia*; in both genera these measure 0.2–0.4 µm in diameter [3, 13, 20, 21]. Such granules have not been observed in x-cells from salmonids [6, present study].

The pellicle of *S. vastator* n. gen., n. sp. and of the coho parasite is markedly electron dense; in *S. vastator* n. gen., n. sp. it was observed that it was composed of several electron-dense membranes, the number of which were often difficult to ascertain but usually there were three. This pellicle could be interpreted as being composed of the plasma membrane with subtending ER cisternae, all with electron-dense membranes. Indeed, what could be the production of such cisternae were seen in a particular cytoplasmic site in some cells, possibly near an (unobserved) Golgi body. In *Gadixcellia gadi* there is a similar three-layered alveolar pellicle (see, for example, Fig. 9 in [14]). *Xcellia* spp. may also show cortical alveolae [5], but in most previous ultrastructural studies of (likely) *Xcellia* spp. cortical ER cisternae were rare or only sparsely distributed (see, for example, Fig. 4 in [22]).

## Conclusions

A particular type of lesions associated with disease in farmed salmonids in Norway are due to a novel x-cell type protist parasite named *Salmoxcellia vastator* n. gen., n. sp. This parasite occurs in systemic infections in rainbow trout and Atlantic salmon, but the liver, heart, kidneys and red musculature are particularly invaded. *Salmoxcellia vastator* n. gen., n. sp*.* occurs as aggregations of multinuclear x-cells that are filled with lipid granules and surrounded by several electron-dense membranes.

Phylogenetic analyses place *S. vastator* n. gen., n. sp. together with xcelliids, a group of similar parasites infecting certain marine fish species. These parasites are perkinsozoan alveolates, and the alveolate affinities of *Salmoxcellia* are further supported by a pellicle composed of flat alveoli subtending the plasma membrane.

## Data Availability

The data supporting the conclusions of this article are included within the article. The sequence alignment used in the phylogenetic analysis is available from the corresponding author on request. The new sequences were submitted to the GenBank database under the accession numbers MW743278–MW743281. Hapantotype material was deposited in the Zoological Museum, University of Bergen under the accession numbers ZMBN 140945 and ZMBN 140946.

## Abbreviations

BLAST: Basic Local Alignment Search Tool
Cq: Cycle threshold value
LSID: Life Science Identifier
rDNA: Ribosomal RNA gene
SSU: Small subunit
TEM: Transmission electron microscopy
