# Correction: A novel protist parasite, *Salmoxcellia vastator* n. gen., n. sp. (Xcelliidae, Perkinsozoa), infecting farmed salmonids in Norway

**DOI:** 10.1186/s13071-022-05510-5

**Published:** 2022-10-14

**Authors:** Egil Karlsbakk, Cecilie Flatnes Nystøyl, Heidrun Plarre, Are Nylund

**Affiliations:** 1 Department of Biological Sciences, University of Bergen, PO Box 7803, 5020 Bergen, Norway; 2 Skodje, Norway

## Correction: Parasites Vectors (2021) 14:431 10.1186/s13071-021-04886-0

Following publication of the article [1], it came to the journal's attention that the following text had been erroneously included in the Gross pathology section of the Results: "ZooBank registration: The LSID for the new genus is XXXX". This redundant text has since been removed from the article.
